# Simplifying Diagnosis of Fetal Alcohol Syndrome Using Machine Learning Methods

**DOI:** 10.3389/fped.2021.707566

**Published:** 2022-01-21

**Authors:** Moritz Blanck-Lubarsch, Dieter Dirksen, Reinhold Feldmann, Eike Bormann, Ariane Hohoff

**Affiliations:** ^1^Department of Orthodontics, University of Münster, Münster, Germany; ^2^Department of Prosthodontics and Biomaterials, University of Münster, Münster, Germany; ^3^Department of Pediatrics, University of Münster, Münster, Germany; ^4^Department of Biostatistics and Clinical Research, University of Münster, Münster, Germany

**Keywords:** fetal alcohol spectrum disorder, 3D facial scans, machine learning, decision tree, K-nearest neighbor, support vector machine

## Abstract

**Introduction:**

The fetal alcohol spectrum disorder (FASD) is a complex and heterogeneous disorder, caused by gestational exposure to alcohol. Patients with fetal alcohol syndrome (FAS—most severe form of FASD) show abnormal facial features. The aim of our study was to use 3D- metric facial data of patients with FAS and identify machine learning methods, which could improve and objectify the diagnostic process.

**Material and Methods:**

Facial 3D scans of 30 children with FAS and 30 controls were analyzed. Skeletal, facial, dental and orthodontic parameters as collected in previous studies were used to evaluate their value for machine learning based diagnosis. Three machine learning methods, decision trees, support vector machine and k-nearest neighbors were tested with respect to their accuracy and clinical practicability.

**Results:**

All three of the above machine learning methods showed a high accuracy of 89.5%. The three predictors with the highest scores were: Midfacial length, palpebral fissure length of the right eye and nose breadth at sulcus nasi.

**Conclusions:**

With the parameters right palpebral fissure length, midfacial length and nose breadth at sulcus nasi, machine learning was an efficient method for the objective and reliable detection of patients with FAS within our patient group. Of the three tested methods, decision trees would be the most helpful and easiest to apply method for everyday clinical and private practice.

## Introduction

The fetal alcohol spectrum disorder (FASD) is a developmental disorder with an estimated worldwide prevalence of 0.77 % (1, 2). It is caused by maternal alcohol intake during pregnancy and results in lifetime problems for the affected person and high costs for the public health care systems (3, 4).

FASD symptoms comprise growth deficiencies, abnormal facial phenotype and damage or dysfunction of the central nervous system. Lange et al. describes correlations between FASD and 428 accompanying diseases (1, 5). In a study concerning daily living skills of adult patients with FASD, the affected persons showed lower skills than IQ-matched controls (6). A meta-analysis by Popova et al. describes that if lacking an appropriate diagnosis, intervention and support, persons affected with FASD are at a high risk for becoming involved in the legal system as offenders or as victims. According to the same meta-analysis, youth with FASD are 19 times more likely to be incarcerated than youth without FASD (2, 5, 7). Streissguth et al. describe higher rates of disrupted school experience, trouble with law, inappropriate sexual behavior, alcohol and drug problems for FASD patients (8).

FASD as a generic term comprises different severities of the disorder. The most severe form is the fetal alcohol syndrome (FAS), followed by the partial fetal alcohol syndrome (pFAS), the alcohol-related birth defects (ARBD) and the alcohol-related neurodevelopmental disorders (ARND) (9).

The different subgroups of FASD are diagnosed within four diagnostic categories, namely (a) growth deficiencies, (b) facial characteristics, (c) abnormalities of the central nervous system and (d) confirmed or unconfirmed intrauterine alcohol exposure. However, across the four most current FASD diagnostic guidelines (4-Digit Code, Canadian, IOM, and Revised IOM), there are different criteria as for the division into the subgroups (FAS, pFAS, ARBD and ARND). For the diagnosis fetal alcohol syndrome (FAS), abnormalities in all four diagnostic categories (a), (b), (c), (d) should be present. However, diagnosis of FAS is still possible, even if exposure to alcohol cannot be verified. This is due to the significant abnormalities of (a), (b), and (c). For the other three less severe forms of FASD, alcohol consumption must be confirmed. The diagnosis pFAS is verified if all characteristics (a–d) are found but less severe, only the Canadian guideline defines pFAS without growth deficiencies (a). For the diagnosis ARND growth deficiencies (a) and facial phenotype (b) are not mentioned in the Canadian and IOM guidelines, for the 4-digit-code only growth deficiencies (a) do not have to be detectable, but all other categories apply. The diagnosis ARBD is a category only present in the IOM and revised IOM guideline and can be verified if the central nervous system (c) is without abnormalities but all other three categories apply (10).

To date it is still challenging to accurately detect patients with FASD (11) as most diagnostic guidelines are based on different methods and are in parts based on subjective evaluation (12).

Studies concerning FAS as the most severe form of FASD found a wide range of craniofacial and dental anomalies, which can be used in the course of the diagnostic process (13–22). The most established guideline for the diagnosis of FASD is the 4-digit diagnostic code, which was developed by Astley et al. (19). The lip-philtrum guide, which is part of the 4-digit diagnostic code, covers the diagnosis of lip and philtrum parameters. The evaluation of the lip and philtrum in patients with FASD as described by Astley et al. is based on comparison with photographs (19, 23, 24), which is a subjective method.

More recent studies by our research group compared 3-D facial and dental cast scans of patients with FAS to healthy control patients. We found significant metric differences concerning vertical facial measurements, philtrum depth, palpebral fissure length, nose breadth, maxillary width and also dental deficiencies (13–18). The aforementioned studies allow for objective metric measurements, which can be used in the diagnostic process of FAS.

Machine learning is a technology, which is presently used in many different areas of everyday life (25). For medical purposes machine learning is becoming of more and more interest for diagnosis and prediction (26). For example, a study on machine learning and diabetic retinopathy detection from photographs showed high sensitivity and specificity for the applied method (27).

Machine learning refers to a class of algorithms that recognize patterns in a training dataset and use them to derive predictions for new input data. In the methods used here, this is done in the form of “supervised learning”, i.e. labels are specified for the training data for the classification to be performed. The evaluation of the data is done by statistical models, the parameters of which are adjusted in the course of the “learning process” by means of regression techniques. These can subsequently be applied to new data sets. These methods include, for example, the algorithms known as “decision trees”, “support-vector machines,” and “k-nearest neighbors” (28).

In the case of the decision trees (DT), the result is a classification based on a sequence of branched yes/no decisions, which are made depending on threshold values determined for the features (29). The support-vector machine (SVM), on the other hand, renders a classification by separating data in the n-dimensional parameter space with a hyperplane, where n denotes the number of features considered. Basically, this is a linear regression technique which tries to maximize the gaps between different classes by paying special attention to the marginal values (30). Finally, the k-nearest neighbors method (KNN) performs the classification by assigning samples according to the distances of the feature values to the most common among its k nearest neighbors (31). For a practical introduction to these methods see (32).

Therefore, machine learning can help the clinician in making a diagnosis based on input data, even if the clinician is not an expert in the particular medical field. For example, machine learning methods, trained by supervised learning can enable the clinician to take metric data of a patient and obtain a prediction on the diagnosis based on known patterns. This, of course requires profound databases with verified values for the known patterns given to the computer during the supervised learning process.

For medical purposes decision trees represent an easy to use method *via* yes/no decisions. If a comparable accuracy for diagnosis can be achieved, decision trees may therefore be preferred over other machine learning methods. In this study, we included decision trees as well as support vector machine and k-nearest neighbors methods to compare the accuracy of these commonly used machine learning techniques.

A number of morphological and medical parameters are known in which children with FAS may differ from their peers. Of these, the following data are available from previous studies for review as for their potential in machine learning based diagnosis:

These parameters in patients with FAS comprise skeletal parameters such as significantly smaller head circumference, facial and dental parameters. The facial parameters are: significantly lower philtrum depth, smaller nose breadth at sulcus nasi, reduction in palpebral fissure length, smaller inner canthal distance (in male children with FAS), shorter middle facial third, longer lower facial third, greater philtrum length, profile more askew to the back in comparison to healthy controls (14, 15).

Dental parameters show higher values for the Developmental Defects of Enamel (DDE)-index and the Decayed Missing Filled Teeth (DMFT)-index, which both are significantly higher in patients with FAS (18).

Orthodontically a study could identify a significant increase in malocclusion (higher Peer Assessment Rating (PAR) score) and a higher prevalence of crossbites in patients with FAS (13).

The aim of our study was to use the existing 3D- metric facial data of patients with FAS and identify machine learning methods, which could improve and objectify the diagnostic process.

## Materials and Methods

### Study Design, Inclusion Criteria and Participants

In the course of previous investigations, 3D-facial scans and scans of dental casts of a total of 30 Caucasian children with FAS (mean age 8.8 years; range 6.6–11.2 years; 15 male and 15 female) and 30 healthy Caucasian controls (mean age 8.2 years; range 5.8–11.9 years; 18 male and 12 female) were taken in the Department of Orthodontics of the University Hospital Muenster in the time period between 2012 and 2016 (13–18). Children with FAS were recruited in cooperation with the Department of Pediatrics of the University Hospital Muenster. The control group consisted of voluntary healthy children from local schools. The FAS diagnosis was verified by a pediatric specialist according to the German FAS diagnostic guideline (3).

### Exclusion Criteria

Patients with less severe forms of FASD such as partial FAS, ARBD or ARND were excluded from our investigation.

Exclusion criteria for both groups were former or present orthodontic treatment, deciduous or permanent dentition and any previous or present disease, trauma, surgical intervention, disorder or syndrome affecting craniofacial structures.

### Facial 3D Scan

**Figure 1:**
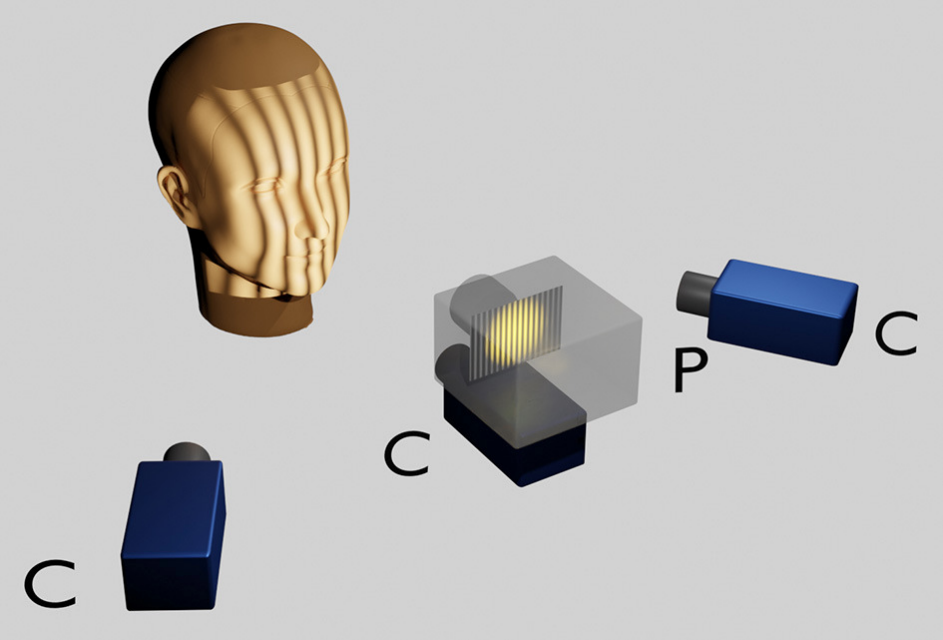
Optical 3D measurement system based on the fringe projection technique. The head of the sitting patient, positioned at a defined distance from the scanner is adjusted according to the Frankfort horizontal and the pupillary planes parallelised to the ground horizontal with the aid of a light projection. A LCD projector in the middle projects a sequence of binary and sinusoidal vertical stripes onto the face, which are recorded by three charge coupled device (CCD) cameras. Within approximately 1 s a point cloud consisting of approximately 50,000–800,000 facial 3D coordinates is rendered per patient.

The face scans were carried out with an optical 3D measurement system based on the fringe projection technique developed at the University Hospital Muenster (33). With an LCD projector (VT 58, NEC), 13 different fringe patterns are projected onto the face and recorded by three cameras (Imagingsource GmbH, Bremen, Germany) with a digital interface (IEEE1394) at a resolution of 1,024 x 768 pixels. The images are evaluated photogrammetrically which renders approx. 50,000 to 800,000 coordinates. The measurement takes about 1.5s (Figure 1).

### Machine Learning Methods

Three different supervised machine learning methods were used and compared concerning applicability in the clinical diagnostic process of FAS:

1. Support vector machine (SVM)
2. K-nearest neighbors (KNN)
3. Decision trees (DT)

The methods in question were implemented in the Python programming language (version 3.7.1) (34) using the Scikit-learn library (version 0.21.3) [https://scikit-learn.org/stable/tutorial/basic/tutorial.html] (35, 36).

For statistical evaluation of the results the statistics software R (Version 3.6.2) (37) was used. All calculations were carried out on a personal computer running on the Microsoft Windows 10 operating system.

All three machine learning techniques perform a classification based on regression techniques using the selected features. For the decision tree (DT), the branched yes/no decisions were based on a criterion called the Gini impurity. This is a measure of the likelihood of an incorrect classification of an element. The Gini impurity varies between 0 and 1, with 0 representing a perfect classification of a yes/ no decision whereas 1 represents a random distribution of features to the yes/no decision. Therefore, Gini should be ideally close to 0.

As a further means to restrict the complexity of the tree, the depth of the tree was limited to two nodes where the minimum sample size required for a split was two [https://towardsdatascience.com/decision-trees-in-machine-learning-641b9c4e8052].

The support vector machine (SVM) on the other hand renders a classification by separating data in the three-dimensional parameter space with a hyperplane.

In the K-Nearest Neighbors method (KNN), the number of neighbors used for classification (k) was set to 5.

### Variables and Data: Calculation of Best-Fitting Predictors

The following demographic and morphometric parameters were included in the evaluation as possible predictors for FAS: Age in months; philtrum depth; palpebral fissure length left eye (PFL l); palpebral fissure length right eye (PFL r); midfacial length (MFL); asymmetry index; palatal depth; mouth breadth; nose breadth at sulcus nasi (NBSN); inner canthal distance.

However, a regression method with so many parameters would make the statistical models too specific to the training data (overfitting). As a means of regularization, i.e., to prevent overfitting, three parameters were selected from the statistical distributions, which showed the greatest discriminatory power between the group of children with FAS and the control group. This was carried out using a scoring approach based on the ANOVA F-value which is the variance of the group means divided by the mean of the within group variances. This means, the morphometric parameters were scored according to their F-values, which is the standard approach used in the Scikit-learn software library.

### Training and Evaluation of Machine Learning Methods

Before the data were classified using these procedures (with the exception of the decision tree), the individual predictors were scaled to ensure the same weighting of all features.

**Figure 2:**
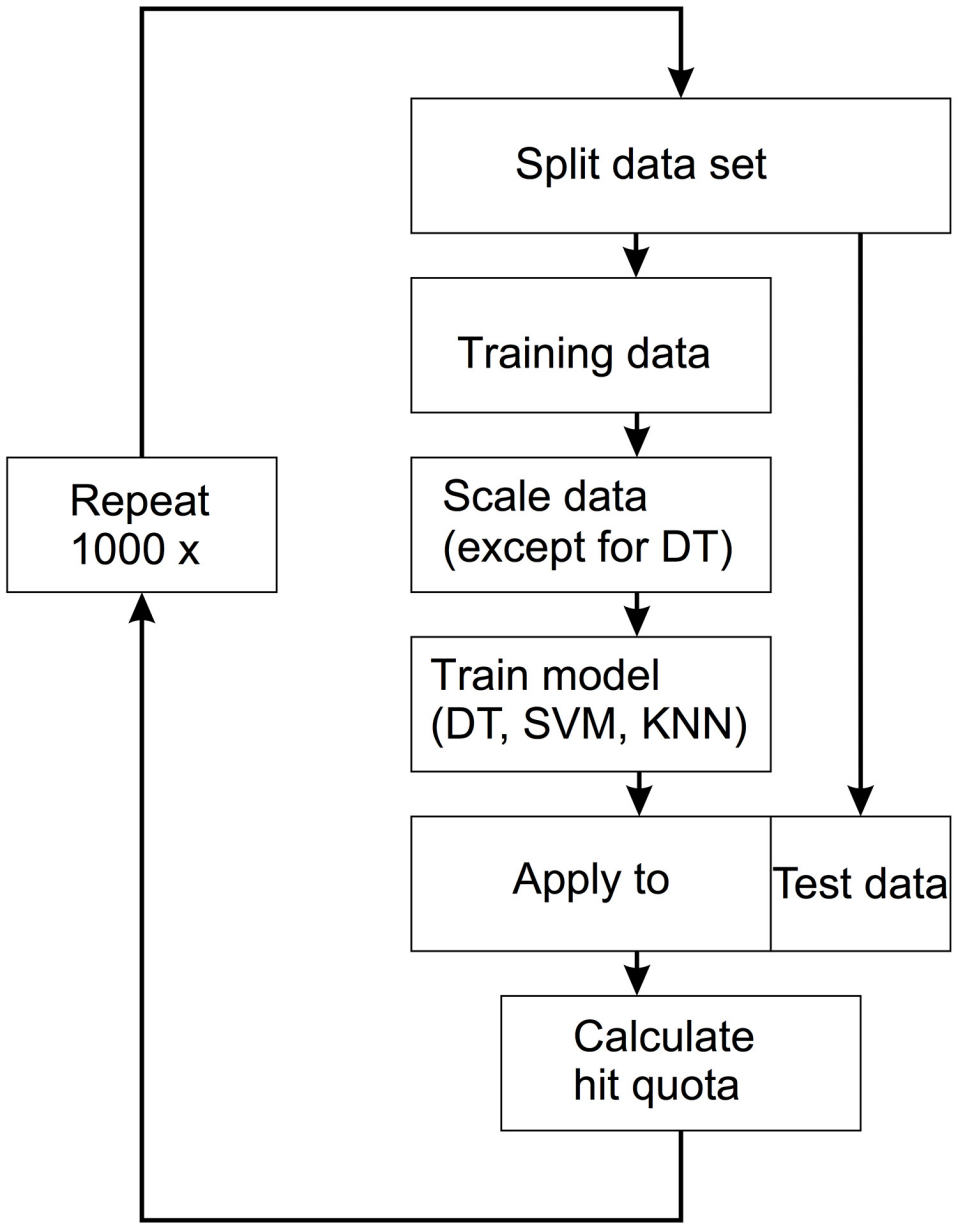
Workflow for the applied supervised machine learning technique. The data set is split into a training set (66% of the data) and a test set (33% of the data). The three models [decision trees (DT), support vector machine (SVM), k-nearest neighbors (KNN)] were then fitted to the training data. The models are then applied to the test data and the hit quota is calculated. This process is repeated 1,000 times.

The machine learning approach used here was supervised learning. This means that all samples were labeled according to whether they belonged to the FAS group or to the controls. The data set was randomly split into a training set (66% of the data) and a test set (33% of the data). The three models (DT, SVM, KNN) were then fitted to the training data. Finally, the models were applied to the test set and the percentage of correctly classified samples was calculated. The procedure was repeated 1,000 times and the distributions of the hit rates of the three methods were determined. The entire flow is shown in Figure 2.

### Bias

To minimize bias, controls were recruited from local schools rather than an orthodontic university department to avoid selection of extreme malocclusions and oral phenotypes, which could have had a potential influence on facial contour. All study participants were screened based on a standardized orthodontic examination protocol and all scans and measurements were performed by the same experienced orthodontist. All data regarding study groups were blinded prior to measurements and statistical evaluation. Since children with FAS show a delayed developmental trajectory, we chose to include slightly (but not statistically significant) younger children as controls in order to optimize comparability (8, 38, 39). Thus, the included children were similar in terms of parameters such as body length or weight.

## Results

### Selection of Predictors

**Figure 3:**
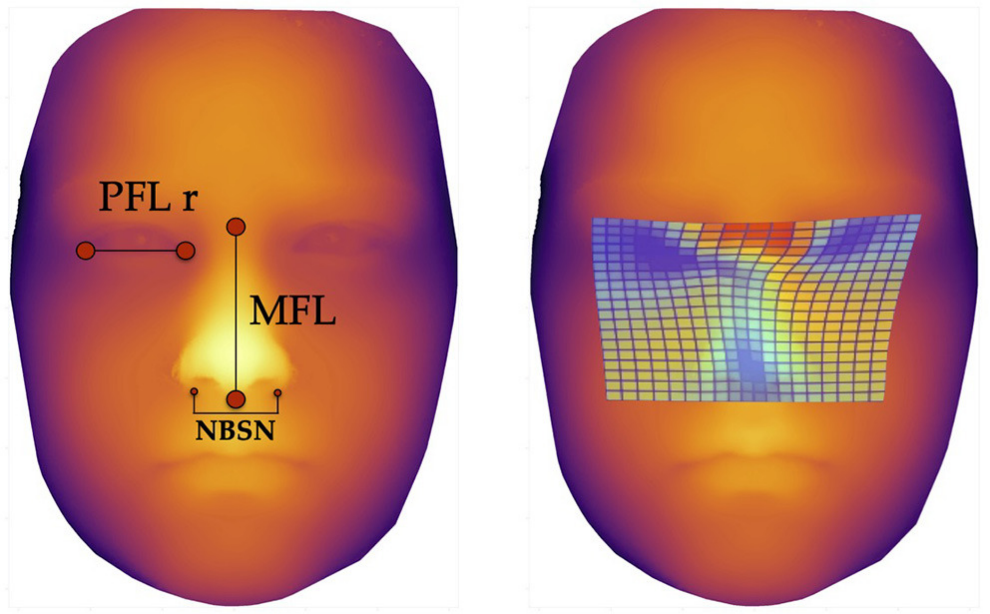
Facial landmarks and thin plate spline visualization. The left image shows the landmarks for measurement of the three predictors for machine learning methods, defined *via* results of the ANOVA-F analysis (PFL r, MFL, and NBSN); the image on the right side shows thin plate spline. These thin plate splines enable visualization of aberrant facial structures. The blue areas show lower distances for palpebral fissure length, midfacial length and nose breadth at sulcus nasi.

The three predictors with the highest scores, i.e., those with the highest F-values were the following facial parameters (Figure 3):

1. Nose breadth at sulcus nasi (NBSN) (F= 60.81) (*p* < 0.001)
2. Midfacial length (MFL) (F= 53.04) (*p* < 0.001)
3. Palpebral fissure length right eye (PFL r) (F= 30.60) (*p* < 0.001)

**Figure 4:**
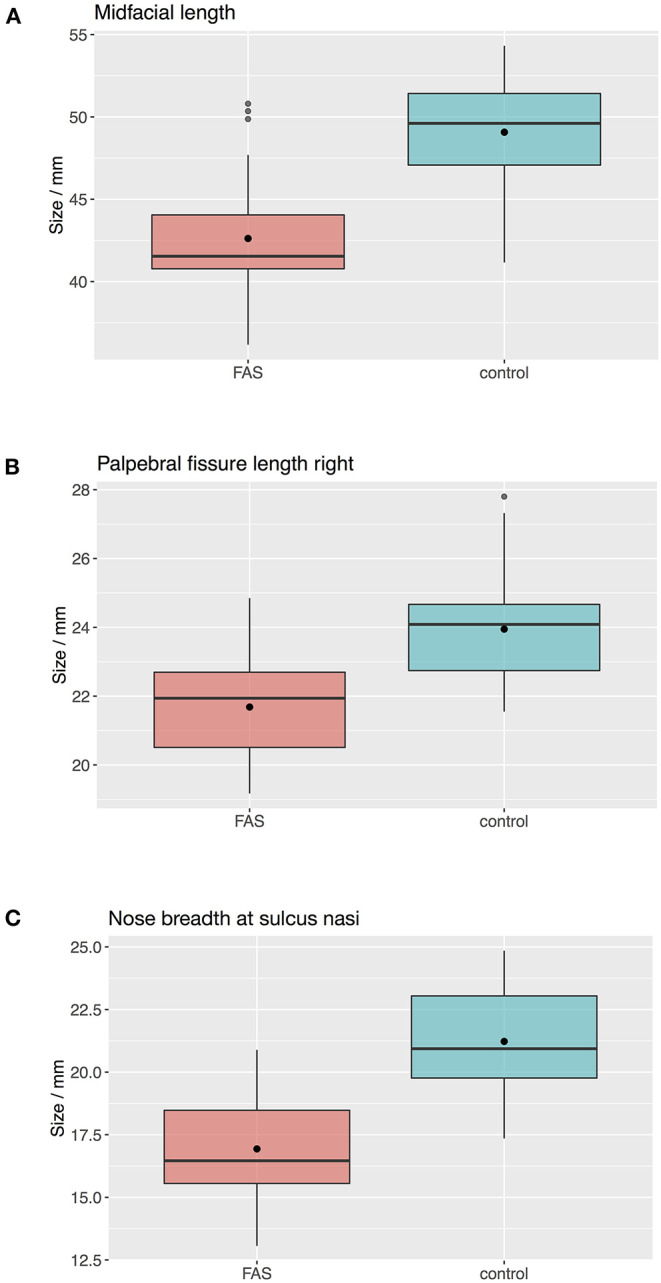
Boxplots for facial landmarks. The boxplots for midfacial length **(A)**, right palpebral fissure length **(B)** and nose breadth at sulcus nasi **(C)** show significant differences between the values for the FAS and control group with lower results for patients with FAS. Nevertheless overlapping of all three parameters for patients with FAS and control group can be found.

**Figure 5:**
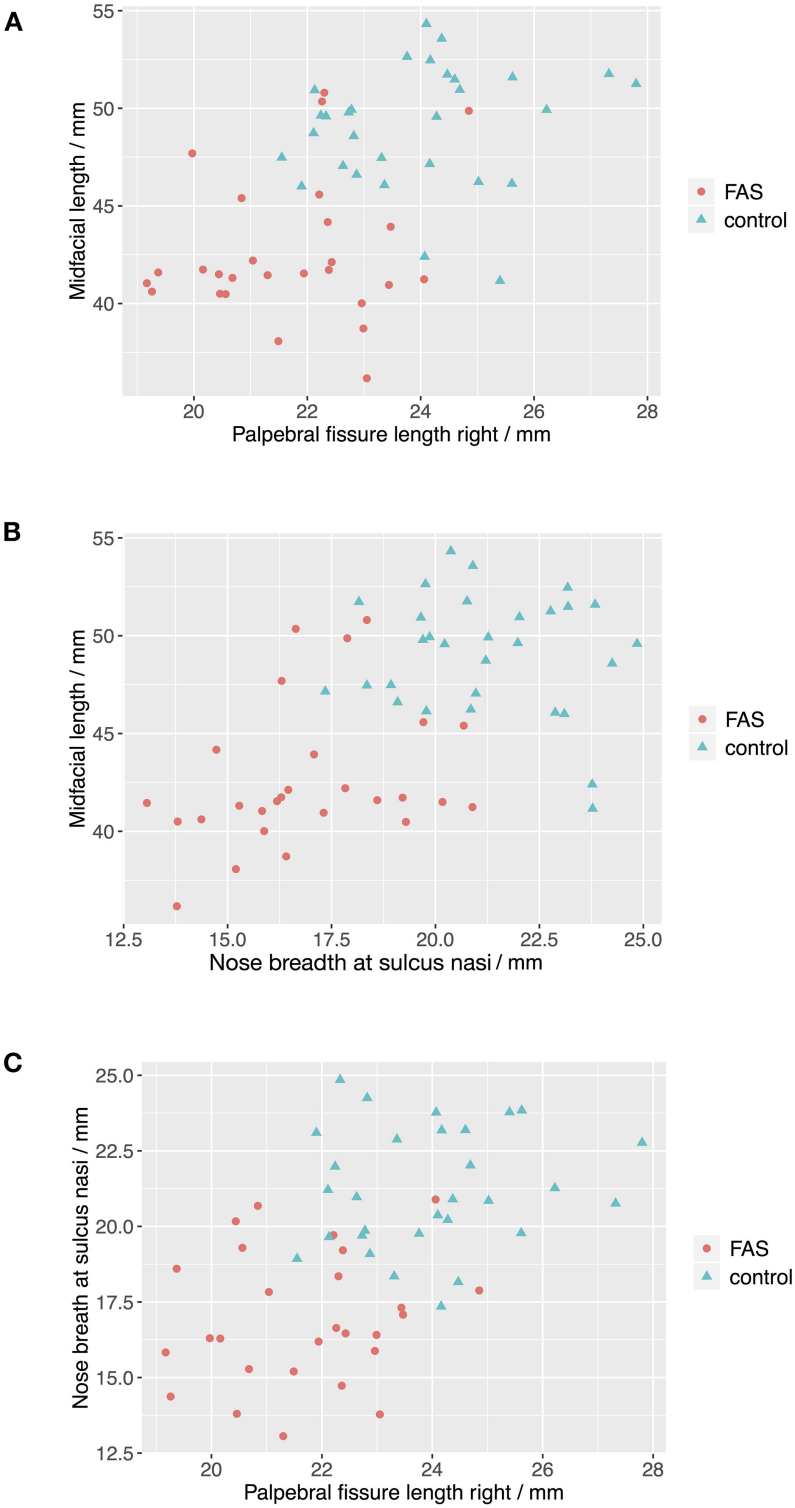
Scatter plots for landmark values. The scatter plots show that a complete division of the values by a trend line is not possible for all combinations of the predictors **(A)** Palpebral fissure length righ vs. midfacial length, **(B)** Nose breath at sulcus nasi vs. midfacial length, and **(C)** Palpebral fissure length right vs. nose breadth at sulcius nasi.

The Boxplots (Figure 4) for midfacial length (Figure 4A), right palpebral fissure length (Figure 4B) and nose breadth at sulcus nasi (Figure 4C) show significant differences between the values for the FAS and control group with lower results for patients with FAS. Nevertheless overlapping of all three parameters for patients with FAS and control group can be found. This overlapping impedes solitary clinical diagnosis, which explains the need for machine learning methods in order to improve the diagnostic accuracy. Figure 5 shows scatter plots for all possible combinations of the three above mentioned predictors. The scatter plots demonstrate that a complete division of the values by a trend line is not possible (Figure 5). Taking these outcomes into account, significant differences for the three predictors could be found. However, a simple classification into patients with FAS vs. healthy controls with conventional methods is not possible. Consequently, these three features were used to compare the three machine learning methods.

### Accuracy of Support Vector Machine

**Figure 6:**
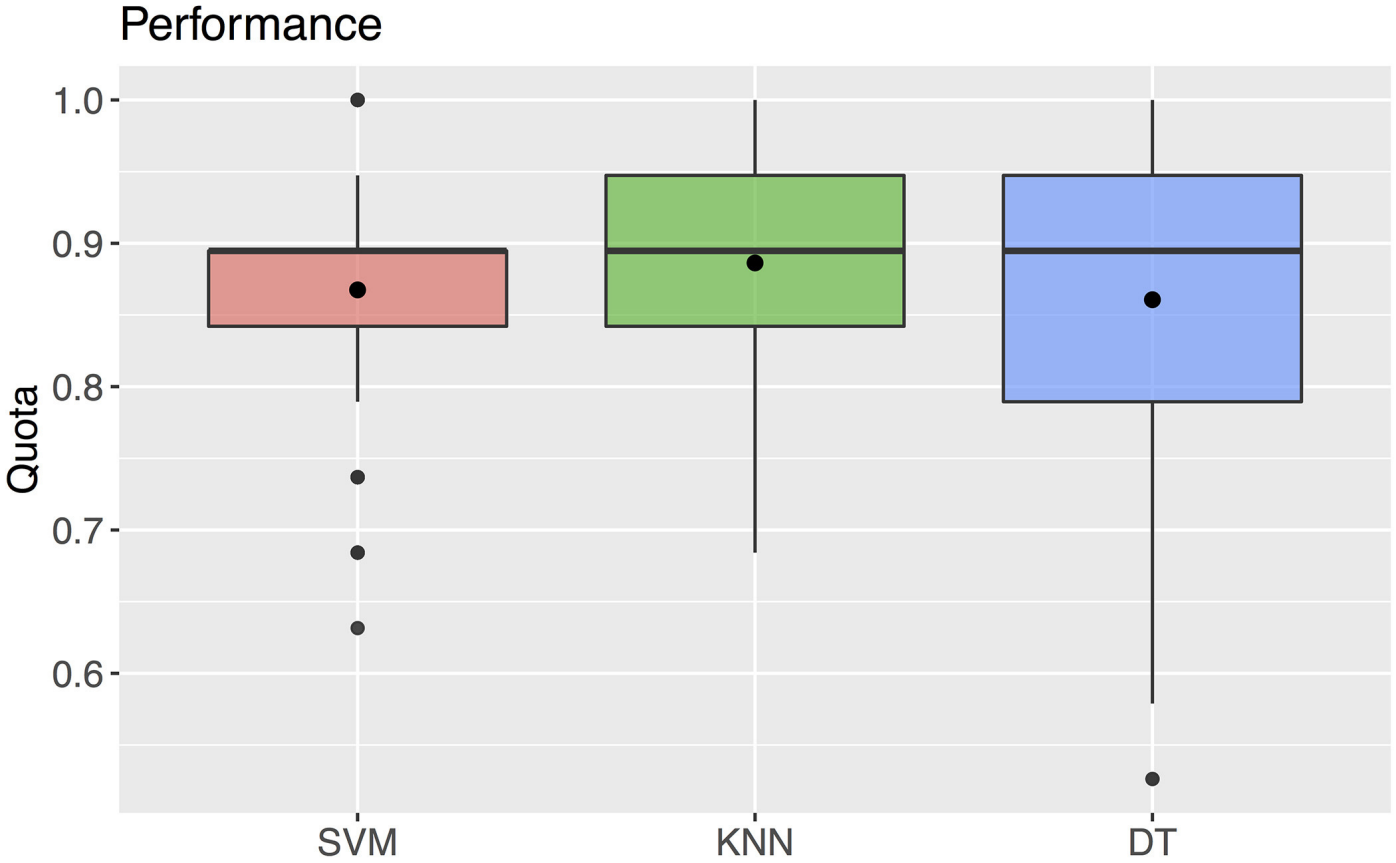
Quota for the three machine learning methods SVM, KNN, DT. The black rectangles denote the mean values. The boxplots show a median of 0.895 for all three parameters.

The support vector machine method resulted in a median predictability of accurate diagnosis of 0.895 (25% quantile 0.842, 75% quantile 0.895) (Figure 6). For this method, the classification, i.e., the diagnosis, was determined by the algorithm using the values of the three predictors selected with the ANOVA F analysis.

### Accuracy of K-Nearest Neighbors

Using the k-nearest neighbors method, the correct diagnosis was possible with a predictability of median 0.895 (25% quantile 0.842, 75% quantile 0.947) (Figure 6). For this method, the values of the three predictors selected with the ANOVA F analysis were also used for classification.

### Accuracy of Decision Tree

**Figure 7:**
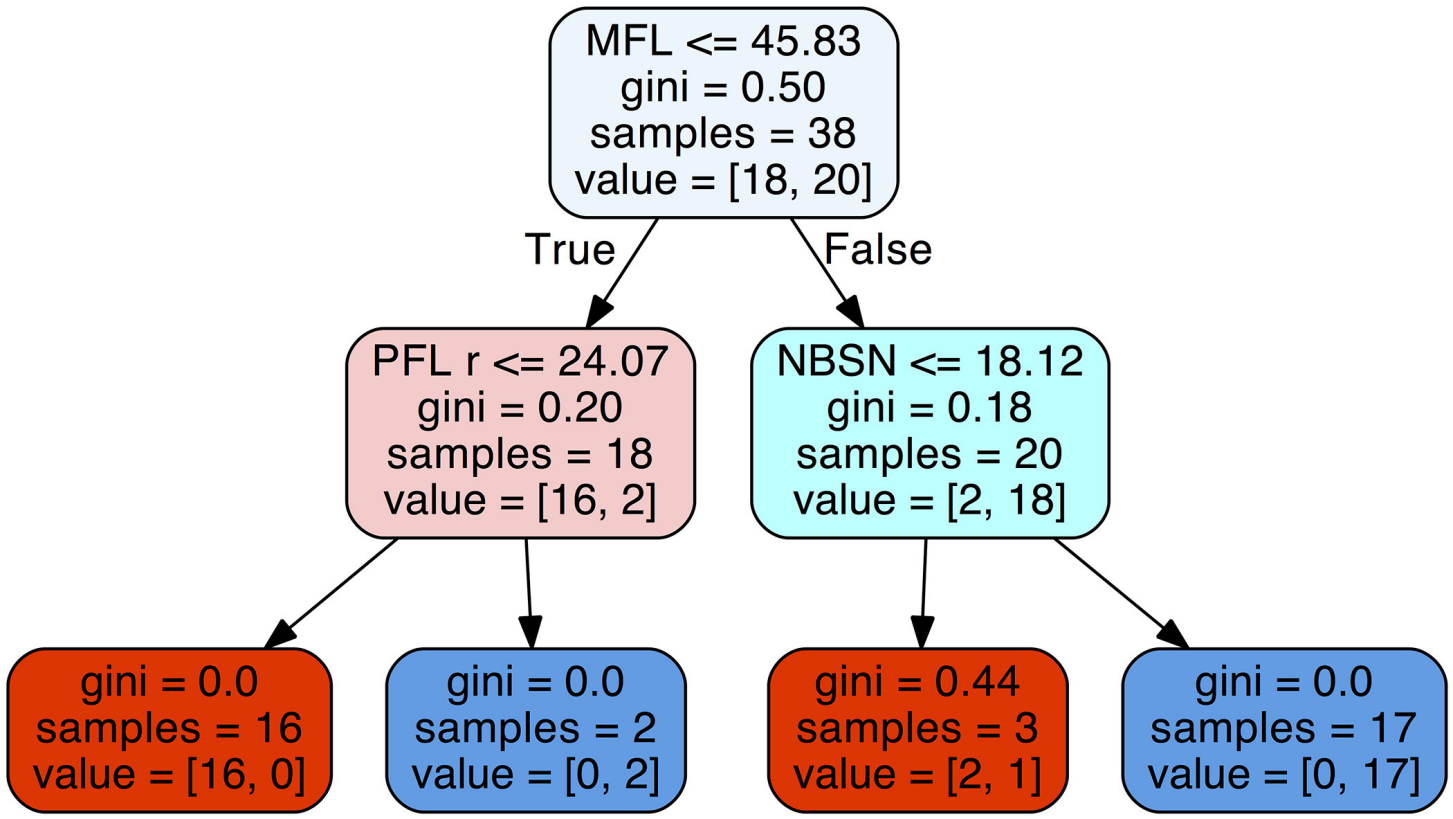
Decision tree for the parameters midfacial length (MFL), right palpebral fissure length (PFL r), and nose breadth at sulcus nasi (NBSN).

The decision tree method resulted in a predictability of a median of 0.895 with 25% quantile 0.790 and 75% quantile 0.947 for FAS diagnosis (Figure 6). For our patient data three metric measurements were necessary (Figure 7):

1) Measurement of the midfacial length. If the value was equal or below 45.83 mm the next step is the measurement of the palpebral fissure length. If the value of the palpebral fissure length is equal or below 24.07 mm the diagnosis is positive for FAS. If the value palpebral fissure length is larger than 24.07 mm, the patient is not affected by FAS.

2) If the measurement of the midfacial length is larger than 45.83 mm the next step is the measurement of nose breadth as sulcus nasi. If the value is equal or below 18.12 mm the patient has FAS. If the value is larger than 18.12 mm, the patient is not affected by FAS.

## Discussion

Up to now the diagnostic process in FASD patients is difficult and some parameters depend on subjective assessment (12). As this leads to a comparatively high number of misdiagnosed cases, there is a strong need for new and improved methods to help diagnose FASD correctly (11).

A study by Goh et al. described a high accuracy of the results when using the decision tree method to identify children affected by prenatal alcohol exposure (40). The parameters used in this study were two parent questionnaires, an IQ score, and a physical examination. However, interviewing biological parents may lead to answers that are not objective or even untrue, and for a majority of these children it is not possible to get answers at all as they live in foster care.

Therefore the use of facial parameters as proposed in our study is a big advantage as they provide reliable data. They can be obtained by taking 3D- scans of intraoral and facial structures which can be done within a short period of time. They have a high accuracy and patients' compliance to this method is very good. The subsequent analysis of the facial and dental features in the 3D-scans can easily be done by the practitioner and is not very time-consuming either.

Recent studies by Suttie et al. 2013 and Valentine at al. 2017 suggest that computer based facial recognition could also be used for the detection of facial features of less severe forms of FASD (such as pFas or ARND) (20, 41). This implies that our findings for aberrant facial structures in patients with FAS (the most severe form) may possibly also be detected in children with more moderate forms of FASD. To date this is not included in the established diagnostic guidelines. Therefore it is important to investigate this option in further studies covering the analysis of facial structures in children with less severe forms of FASD.

Machine learning methods are already used in many fields. Zhang et al. compared machine learning methods (decision trees, support vector machines and k- nearest neighbors) for the detection of multiple sclerosis in magnetic resonance imaging. They could show best results for the use of the k-nearest neighbors method (42). This is in accordance with our results for the k-nearest neighbors method, which showed the highest accuracy with the smallest standard de*via*tion (m = 0.886; SD = 0.059).

All three machine learning methods tested in our study showed high accuracy with *m* > 0.8. However, we found that the decision tree method is the most suitable approach for clinical practice. Using this method, medical staff can easily diagnose FAS patients *via* simple yes/no decisions.

A limitation of this study is that metric results for the yes/no decisions in the decision tree method were based on values from earlier studies with group sizes of ~30 Caucasian children each. In further studies they should be extended to other racial groups as well, as values for facial features can differ. Our study population includes children at primary school age since difficulties associated with FASD often become evident for the first time at the beginning of primary schooling and lead to the consultation of a specialist. According to May et al., this age group is also best suited for an accurate diagnosis as most physical, behavioral and neuropsychological signs are sufficiently evident and verifiable. Of course, the earlier FASD is diagnosed, the higher is the benefit for treating the affected children, which supports the need for early detection methods (43).

The described ANOVA F statistics is a valuable method for the evaluation of suitable parameters, which should be used for the choice of parameters from 3D facial values in bio-databases. The ANOVA F value of our data suggested that the three parameters described above [midfacial length (MFL), right palpebral fissure length (PFL r), nose breadth at sulcus nasi (NBSN)] were the most suitable ones for detecting FAS. This might be different for larger patient samples or different age groups.

Further studies and a larger number of values for the suggested facial or dental parameters are necessary in order to develop a decision tree which could be used in clinical practice.

Research done in this paper is just a start and needs to be repeated with larger groups of patients at different ages and of different racial background and should be extended to the other subgroups of FASD as well.

A strength of this study are the clear results for all three machine learning systems, even though the group size was small. This shows the efficiency of machine learning methods for the diagnosis of FAS patients in general. Decision trees in particular stand out as being a new efficient and easy to apply method.

In the future, decision trees could be implemented in everyday clinical practice and could simplify diagnostic process *via* yes/no decisions after metric measurements. Furthermore even pediatricians or general practitioners could use the decision tree method to check a suspected diagnosis for FASD. This would lead to earlier referral to a specialist and therefore earlier diagnosis and help for the affected patient.

## Conclusion

Within our study sample, machine learning in combination with the parameters palpebral fissure length, midfacial length and nose breadth at sulcus nasi proved to be an efficient method for the objective and reliable detection of patients with FAS. Machine learning can help the clinician in making a preliminary diagnosis based on morphometric data. This, of course requires substantial databases with verified values for the known patterns given to the computer during the supervised learning process.

Clinically, the decision tree method is the simplest and least time consuming method for the detection of FAS.

## Data Availability Statement

The raw data supporting the conclusions of this article will be made available by the authors, without undue reservation.

## Ethics Statement

The studies involving human participants were reviewed and approved by Ethics Committee of the Medical Association of Westphalia – Lippe and the Department of Medicine, University of Münster, Germany, study-code 2012-196-f-S. Written informed consent to participate in this study was provided by the participants' legal guardian/next of kin.

## Author Contributions

DD, MB-L, and AH contributed to the conception and design of the study. MB-L, RF, and AH performed the data acquisition. Data analysis was performed by MB-L, DD, and EB. The methodology was designed by MB-L, DD, EB, and AH. Software handling was done by DD. MB-L, DD, EB, and AH contributed to the interpretation of the data. MB-L and DD wrote the original draft. All authors contributed to manuscript revision, read, and approved the submitted version.

## Conflict of Interest

The authors declare that the research was conducted in the absence of any commercial or financial relationships that could be construed as a potential conflict of interest.

## Publisher's Note

All claims expressed in this article are solely those of the authors and do not necessarily represent those of their affiliated organizations, or those of the publisher, the editors and the reviewers. Any product that may be evaluated in this article, or claim that may be made by its manufacturer, is not guaranteed or endorsed by the publisher.
